# SARS-CoV-2 infection and COVID19 vaccination across eight immune-mediated inflammatory disorders: A prospective, real-life Belgian cohort study – the BELCOMID study

**DOI:** 10.3389/fimmu.2023.1126351

**Published:** 2023-03-01

**Authors:** Jeroen Geldof, Marie Truyens, João Sabino, Marc Ferrante, Jo Lambert, Hilde Lapeere, Tom Hillary, An Van Laethem, Kurt de Vlam, Patrick Verschueren, Elizaveta Padalko, Triana Lobaton, Séverine Vermeire

**Affiliations:** ^1^ Ghent University Hospital, Department of Gastroenterology and Hepatology, Ghent, Belgium; ^2^ Ghent University, Department of Internal Medicine and Pediatrics, Ghent, Belgium; ^3^ University Hospitals Leuven, Department of Gastroenterology and Hepatology, Leuven, Belgium; ^4^ KU Leuven, Translational Research in Gastrointestinal Disorders (TARGID), Department of Chronic Diseases and Metabolism (CHROMETA), Leuven, Belgium; ^5^ Ghent University Hospital, Department of Dermatology, Ghent, Belgium; ^6^ University Hospitals Leuven, Department of Dermatology, Leuven, Belgium; ^7^ University Hospitals Leuven, Department of Rheumatology, Leuven, Belgium; ^8^ Ghent University Hospital, Department of Laboratory Medicine, Ghent, Belgium; ^9^ Ghent University, Department of Diagnostic Sciences, Ghent, Belgium

**Keywords:** SARS-CoV-2, COVID19, IMID, biologic, immunomodulator, real-world data

## Abstract

**Background:**

The risks and impact of COVID19 disease and vaccination in patients with Immune Mediated Inflammatory Diseases (IMID) remain incompletely understood. IMID patients and particularly patients receiving immunosuppressive treatment were excluded from the original, registrational phase-3 COVID19 vaccination efficacy and safety trials. Real-world observational data can help to fill this gap in knowledge. The BELCOMID study aims to explore the interaction between IMIDs, immune-modulating treatment modalities and SARS-CoV-2 infection and vaccination in a real-life patient cohort.

**Methods:**

A multidisciplinary, prospective, observational cohort study was set up. Consecutive patients with IMIDs of the gut, joints and skin followed at two high-volume referral centers were invited. Both patients under conventional treatment or targeted immune modulating therapies were included. Patient data and serological samples were collected at 3 predefined periods (before COVID19 vaccination, before booster vaccination, after booster vaccination). Primary endpoints were positive PCR-test and SARS-CoV-2 serology reflecting previous SARS-CoV-2 infection or vaccination. Associations with IMID treatment modality and IMID disease activity were assessed. Results of the first two inclusion periods (before booster vaccination) are reported.

**Results:**

At the first inclusion period data was assessed of 2165 IMID-patients before COVID19 vaccination. At the second inclusion period, data of 2065 patients was collected of whom 1547 had received complete baseline COVID19 vaccination and 222 were partially vaccinated. SARS-CoV-2 infection rate remained low in both groups. No significant increase in IMID flare-up rate was noted in patients with prior SARS-CoV-2 infection. Multiple logistic regression analyses did not show a significant influence of IMID-treatment modality or IMID activity on SARS-CoV-2 infection risk (based on PCR positivity or N-serology). Patients treated with conventional immunomodulators, systemic steroids, and patients on advanced therapies such as biologics or small molecules, had reduced S-antibody seroconversion. S-antibody response was also lower in patients without prior SARS-CoV-2 infection and in active smokers. A subset of patients (4.1%) had no S- nor N-antibody seroconversion following complete baseline vaccination.

**Conclusion:**

The BELCOMID study results confirm the benign course of COVID19 infection and vaccination in a large real-life IMID-population. However, our results underscore the need for repeated vaccination and smoking cessation in patients with IMIDs treated with immune-modulating therapies or systemic steroids during the pandemic.

## 1 Introduction

As the severe acute respiratory syndrome coronavirus 2 (SARS-CoV-2) pandemic is turning into a significant wrinkle in modern healthcare history, the exact risks and impact of COVID19 on patients with Immune Mediated Inflammatory Diseases (IMIDs) remain unclear.

SARS-CoV-2 infection may remain paucisymptomatic in up to 80% of individuals. However, COVID19 can progress to acute respiratory distress due to a cytokine storm characterized by massive production of inflammatory cytokines (1, 2). This life-threatening disease phenotype is associated with elderly age and certain comorbidities such as obesity, diabetes and arterial hypertension (1–4). Furthermore, it became clear that COVID19 can present with various extrapulmonary symptoms including gastrointestinal, dermatological and rheumatologic manifestations amongst many others (5–9).

IMIDs reflect a spectrum of disorders of the gut (Crohn’s disease, ulcerative colitis), joints (rheumatoid arthritis, psoriatic arthritis, spondylarthritis) and skin (psoriasis, hidradenitis suppurativa, atopic dermatitis). IMIDs are believed to originate from an inappropriate immune response to environmental triggers in genetically susceptible hosts. Their management has been revolutionized in the past two decades by anti-cytokine therapies, T- and B-cell targeting therapies and most recently, small molecules such as Janus Kinase inhibitors (JAKi) and PDE4 inhibitor apremilast. As the risk of infections is generally considered higher in patients under Targeted Immune-Modulating Therapies (TIMT), this could result in patients mistakenly stopping their medication, putting them at risk for flare, requiring steroid treatment and even hospitalization. Now that vaccines have been developed, it is unclear whether TIMT, and broader immunosuppression, interfere with SARS-CoV-2 serologic responses as IMID-patients were excluded from the original, registrational phase 3 COVID19 vaccine efficacy and safety studies (10–12).

As a consequence, many questions remain regarding the impact of COVID19 infection and vaccination in patients with IMIDs. We therefore explored the interaction between IMIDs, their immune modulating treatment modalities and SARS-CoV-2 in a large and real-life patient cohort.

## 2 Methodology

### 2.1 Population and design

(Detailed description of study methodology in **Supplementary Materials**.)

In March 2020, a cross-disciplinary consortium was set up between the University Hospitals of Leuven and Ghent. Within this consortium a multidisciplinary, prospective, observational cohort study; BELCOMID, was developed. The study was approved by the ethics committee of both hospitals (BC-08030/S64422).

Consecutive patients with IMIDs of the gut, joints and skin seen at the two hospitals were invited to participate between December 17^th^ 2020 and February 28^th^ 2021. Both patients under conventional treatment or TIMT were included. Conventional treatment comprised therapies without immunomodulatory effect (N-IM) and immunomodulators (IMM). N-IM included mesalazine, sulfasalazine, acitretin, metformin, zinc, antibiotics, topical treatment options and light therapy. IMM included methotrexate, ciclosporin, dimethyl fumarate, mycophenolate mofetil, leflunomide, hydroxychloroquine and thiopurines. TIMT options included biologics, JAKi and apremilast.

The study goal was twofold. The initial aim was to explore the association between COVID19 and IMIDs in a large, real-life population. This included prospective analysis of exposure to and infection with SARS-CoV-2 and relating this information to the underlying IMID disease course and treatment modalities within the study population. Secondly, as national vaccination campaigns started, the response to COVID19 vaccination in these patients depending on their different treatment modalities was studied in the same cohort.

Both clinical patient data and serial blood samples were collected at predefined registration periods between December 2020 until February 2022 with a time interval of at least 4 months between sequential sampling (see **Supplementary Figure 1**). Sampling period 1 ran from 17/12/2020 to 28/02/2021, prior to the national vaccination campaign. Period 2 from 01/07/2021 to 24/09/2021, prior to the start of booster vaccinations.

Data on vaccination date and type were verified by check of the Flemish database Vaccinet.

### 2.2 Serologic testing

For SARS-CoV-2 serologic testing, the Abbott Architect™ SARS-CoV-2 immunoglobulin G (IgG) assay (>1.4=positive) was used to detect anti-nucleocapsid antibodies (N-antibodies) and the Abbott Architect™ SARS-CoV-2 IgGII Quant assay (≥50AU/mL=seroconversion) was used to detect anti-spike protein antibodies (S-antibodies) (13, 14). Blood samples of vaccinated patients in whom no seroconversion for S- nor N-antibodies was found, were double checked with the highly sensitive and specific LIAISON^®^SARS-CoV-2 TrimericS IgG assay (≥33.8BAU/mL=seroconversion) (15).

### 2.3 Endpoints

First we explored prevalence of SARS-CoV-2 infection and serologic outcome of vaccination in our IMID cohort. Primary endpoints were therefore: positive PCR test and SARS-CoV-2 serology reflecting previous SARS-CoV-2 infection or vaccination. As a second step we explored potential associations between infection and vaccination with IMID treatment modality, IMID disease activity (using validated disease activity scores) and increased SARS-CoV-2 exposure risk. Last but not least, we performed subgroup analysis on the cohort of patients who seemed to mount a lower antibody response.

### 2.4 Data collection and statistics

All data was collected in a pseudonymized electronic case report form using REDCap^®^ software. For descriptive statistics, data was exported to SPSS Statistics version 27. Analyses were performed in R version 4.0.2 with support of the Ghent University Biostatistics Unit.

Both marginal and conditional associations were examined. Marginal associations were tested using two-sided Pearson’s chi-squared tests, not taking into account potential confounders. Conditional effects were tested using adjusted binary logistic regression models and multiple logistic regression models. Due to the limited number of expected events, the number of explanatory variables that could be included in the binary logistic regression models of positive PCR test and high SARS-CoV-2 serology was restricted. Hence, to test the conditional effect of IMID treatment modality in the total population, the binary logistic regression models were adjusted for the propensity score of the respective treatment, increased exposure risk and BMI category. The propensity score was estimated by fitting a logistic regression model where treatment was the response and potential confounders were the predictors. Potential confounders of the association between treatment modality and positive PCR test or SARS-CoV-2 serology status were considered to be age, gender, smoking status, exposure risk, BMI category, IMID type, comorbidities and vaccination status.

For the continuous endpoint of S-antibody titer, linear regression analyses were performed.

All hypothesis testing was performed at the two-sided 5% significance level. No adjustment for multiple testing was made as the analyses are considered to be exploratory and hypothesis-generating. Results should be interpreted carefully and should be confirmed by other research.

## 3 Results

Results of data collection before the start of the national vaccination campaign (Inclusion period 1) and after onset of the vaccination campaign but before booster vaccination (Inclusion period 2) are presented.

### 3.1 Demographics

At baseline, 2165 IMID-patients participated. At the second inclusion period, data was collected from 1895 (87.5%) of 2165 initial patients and of 170 additional patients. Demographics were comparable for both inclusion periods (**Table 1**). Extensive description of IMID treatments can be found in **Supplementary Table 1**. Treatment patterns were comparable in both periods.

**Table 1:** Demographics.

		Registration period 1 = Before national vaccination	Registration period 2 = Before booster vaccination
**N**		2165	2065
**Gender**	Male/Female	1098/1058 (50.7/48.9%)	1094/969 (53.0/46.9%)
**Age**	<40years old	791 (36.5%)	688 (33.3%)
	>/= 40 - </= 60 years old	816 (37.7%)	737 (35.7%)
	> 60 years old	427 (19.7%)	405 (19.6%)
**IMID type**	IBD	1344 (62.1%)	1340 (65.0%)
	Crohn’s disease	842 (38.9%)	806 (39.0%)
	Ulcerative colitis	444 (20.5%)	422 (20.4%)
	IBD type unclassified	13 (0.6%)	9 (0.4%)
	Rheumatologic IMID	505 (23.3%)	379 (18.4%)
	Rheumatoid arthritis	262 (12.1%)	219 (10.6%)
	Spondyloarthritis	127 (5.9%)	74 (3.6%)
	Psoriatic arthritis	75 (3.7%)	65 (3.1%)
	Dermatologic IMID	316 (14.6%)	346 (16.8%)
	Hidradenitis suppurativa	36 (1.7%)	31 (1.5%)
	Psoriasis	232 (10.7%)	237 (11.5%)
	Atopic dermatitis	33 (1.5%)	28 (1.4%)
**Smoking status**	Active smoker	367 (17.0%)	323 (15.6%)
	No smoking	1469 (67.9%)	1369 (66.3%)
**BMI**	Underweight (<18.5kg/m^2^)	54 (2.5%)	46 (2.2%)
	Normal (≥18, <25kg/m2)	814 (37.6%)	693 (33.6%)
	Overweight (≥25, <30kg/m2)	697 (32.2%)	591 (28.6%)
	Obese (≥30kg/m2)	376 (17.4%)	326 (15.8%)
**Comorbidities**	Heart disease	204 (9.4%)	215 (10.4%)
	Pulmonary disease	65 (3.0%)	67 (3.2%)
	Chronic renal disease	50 (2.3%)	55 (2.7%)
	Chronic liver disease	78 (3.6%)	108 (5.2%)
	Diabetes	99 (4.6%)	90 (4.4%)
	HIV/AIDS	2 (0.1%)	2 (0.1%)
	No comorbidities	573 (26.5%)	488 (23.6%)
**SARS-CoV-2 exposure risk**	Considered increased*	1036 (47.9%)	791 (38.3%)
**IMID treatment modality**	TIMT	1578 (72.9%)	1552 (75.2%)
	IMM	477 (22.0%)	395 (19.1%)
	N-IM	114 (5.3%)	82 (4.0%)
	Combination TIMT/IMM	264 (12.2%)	248 (12.0%)
	Systemic steroids	232 (10.7%)	118 (5.7%)

*SARS-CoV-2 exposure risk was considered increased based on patients’ job description, recent travelling history or potential COVID-19 contacts at healthcare facilities.

### 3.2 COVID19 in IMID-patients before onset of the vaccination campaign

Before onset of the vaccination campaign, almost half of participants (47.9%) were considered to have increased exposure risk to SARS-CoV-2, based on their job-related COVID19-exposure, social contacts and/or shielding behavior.

Symptoms suggestive of COVID19 (irrespective of confirmed infection) were reported by 395 (18.2%) participants. This followed the Belgian epidemiological curve (**Supplementary Figure 2**) (16). Hospitalization for respiratory symptoms was required in 28 patients (1.4%) of whom only one required intensive care unit (ICU) admission for invasive ventilation.

**Figure 1:**
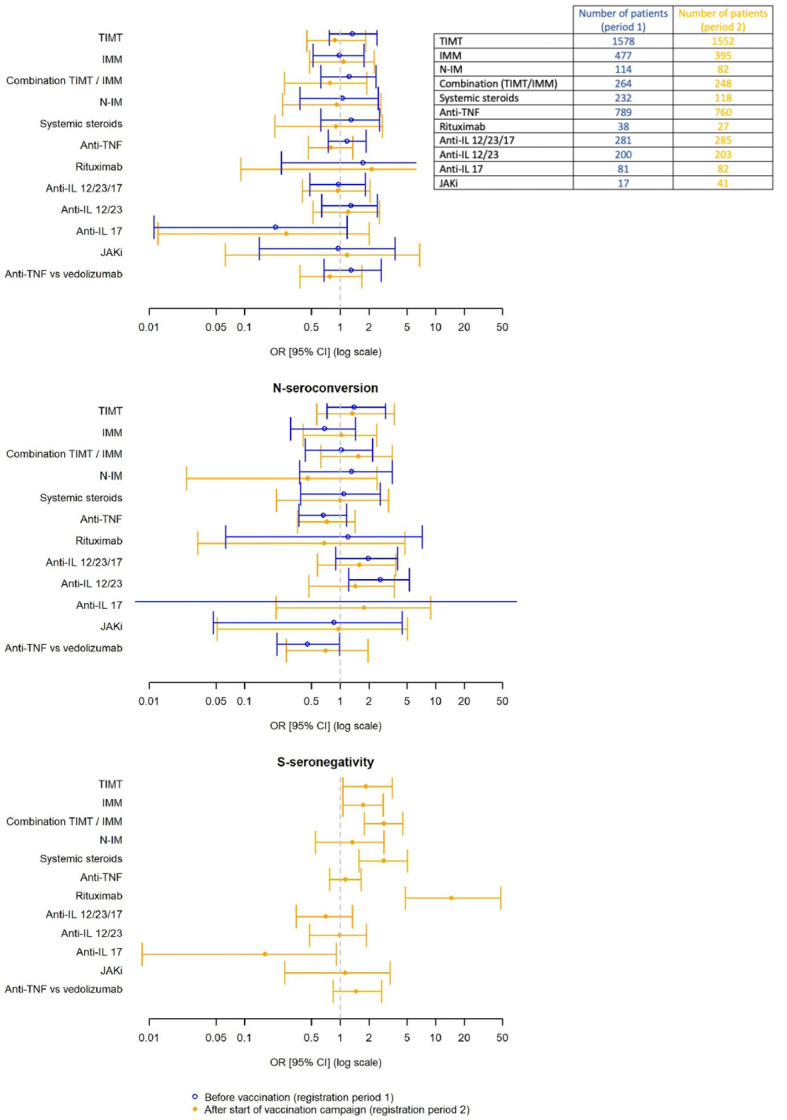
Associations between SARS-CoV-2 PCR/serology and IMID treatment modality.

A total of 104 (5.1%) patients reported a confirmed SARS-CoV-2 infection based on positive nasopharyngeal PCR test. PCR-positivity rate was 9.9% (104/1045 tested patients). PCR-positivity rate was not influenced by flare-up rate of the underlying IMID nor by IMID treatment modality in multiple logistic regression analysis (**Table 2** **/** **Figure 1**).

**Table 2:** Associations* between SARS-CoV-2 PCR/serology and IMID treatment modality.

Outcome parameter	Treatment modality	Before vaccination (registration period 1)	After start of vaccination campaign (registration period 2)
**Positive PCR**	TIMT	OR 1.34, 95% CI 0.77-2.43, P=0.32	OR 0.88, 95% CI 0.45-1.85, P=0.72
	IMM	OR 0.98, 95% CI 0.52-1.77, P=0.95	OR 1.09, 95% CI 0.48-2.27, P=0.82
	Combination TIMT/IMM	OR 1.25, 95% CI 0.63-2.36, P=0.51	OR 0.78, 95% CI 0.26-1.9, P=0.62
	N-IM	OR 1.06, 95% CI 0.38-2.51, P=0.90	OR 0.92, 95% CI 0.25-2.66, P=0.89
	Systemic steroids	OR 1.31, 95% CI 0.63-2.56, P=0.45	OR 0.91, 95% CI 0.21-2.77, P=0.88
	Anti-TNF	OR 1.18, 95% CI 0.75-1.86, P=0.47	OR 0.80, 95% CI 0.46-1.35, P=0.40
	Rituximab	OR 1.74, 95% CI 0.24-8.00, P=0.51	OR 2.14, 95% CI 0.09-20.3, P=0.55
	Anti-IL 12/23 + 23 + 17	OR 0.97, 95% CI 0.48-1.83, P=0.92	OR 0.95, 95% CI 0.40-2.05, P=0.90
	Anti-IL 12/23 + anti-IL23	OR 1.29, 95% CI 0.64-2.45, P=0.45	OR 1.21, 95% CI 0.52-2.57, P=0.63
	Anti-IL 17	OR 0.21, 95% CI 0.01-1.18, P=0.15	OR 0.27, 95% CI 0.01-2.01, P=0.28
	JAKi	OR 0.96, 95% CI 0.14-3.73, P=0.96	OR 1.18, 95% CI 0.06-6.76, P=0.88
	Anti-TNF *vs.* vedolizumab	OR 1.31, 95% CI 0.67-2.69, P=0.44	OR 0.79, 95% CI 0.38-1.68, P=0.52
**N-seroconversion**	TIMT	OR 1.41, 95% CI 0.73-2.98, P=0.33	OR 1.34, 95% CI 0.57-3.69, P=0.54
	IMM	OR 0.69, 95% CI 0.30-1.45, P=0.35	OR 1.03, 95% CI 0.41-2.41, P=0.95
	Combination TIMT/IMM	OR 1.03, 95% CI 0.43-2.19, P=0.94	OR 1.55, 95% CI 0.63-3.50, P=0.32
	N-IM	OR 1.32, 95% CI 0.38-3.51, P=0.62	OR 0.46, 95% CI 0.02-2.43, P=0.46
	Systemic steroids	OR 1.1, 95% CI 0.39-2.62, P=0.84	OR 1.00, 95% CI 0.22-3.20, P=1
	Anti-TNF	OR 0.67, 95% CI 0.37-1.17, P=0.17	OR 0.73, 95% CI 0.36-1.43, P=0.37
	Rituximab	OR 1.21, 95% CI 0.06-7.21, P=0.86	OR 0.68, 95% CI 0.03-4.74, P=0.74
	Anti-IL 12/23 + 23 + 17	OR 1.97, 95% CI 0.90-3.99, P=0.074	OR 1.59, 95% CI 0.58-3.81, P=0.33
	Anti-IL 12/23 + anti-IL23	OR 2.64, 95% CI 1.22-5.31, **P=0.009**	OR 1.44, 95% CI 0.47-3.67, P=0.48
	Anti-IL 17	OR 7.1e-7, 95% CI 6e-84-1260, P=0.98	OR 1.79, 95% CI 0.21-8.89, P=0.53
	JAKi	OR 0.87, 95% CI 0.05-4.45, P=0.89	OR 0.96, 95% CI 0.05-5.08, P=0.97
	Anti-TNF *vs.* vedolizumab	OR 0.46, 95% CI 0.22-0.98, **P=0.04**	OR 0.71, 95% CI 0.27-1.95, P=0.48
**No S-seroconversion**	TIMT	–	OR 1.87, 95% CI 1.07-3.50, **P=0.038**
	IMM	–	OR 1.75, 95% CI 1.07-2.81, **P=0.022**
	Combination TIMT/IMM	–	OR 2.86, 95% CI 1.78-4.54, **P<0.001**
	N-IM	–	OR 1.34, 95% CI 0.55-2.87, P=0.49
	Systemic steroids	–	OR 2.88, 95% CI 1.57-5.08, **P<0.001**
	Anti-TNF	–	OR 1.14, 95% CI 0.78-1.66, P=0.5
	Rituximab	–	OR 14.6, 95% CI 4.80-48.2, **P<0.001**
	Anti-IL 12/23 + 23 + 17	–	OR 0.71, 95% CI 0.35-1.34, P=0.32
	Anti-IL 12/23 + anti-IL23	–	OR 0.99, 95% CI 0.48-1.88, P=0.98
	Anti-IL 17	–	OR 0.16, 95% CI 0.01-0.91, P=0.096
	JAKi	–	OR 1.13, 95% CI 0.26-3.33, P=0.85
	Anti-TNF *vs.* vedolizumab	–	OR 1.47, 95% CI 0.84-2.71, P=0.19

*Multiple logistic regression analysis with adjustment for propensity score, exposure risk and BMI category. Bold means to highlight statistically significant results (this is the case if P<0.05).

N-antibody seroconversion and thus confirmed SARS-CoV-2 infection, was found in 3.2% of patients before vaccination. N-antibody seroconversion rate was significantly higher in patients with increased COVID19 exposure risk (RR=3.05, 95%CI 1.80-5.19, P<0.001) and in patients with prior positive PCR test (RR=62.25, 95%CI 28.92-133.96, P<0.001).

Multiple logistic regression analysis did not show significant associations between N-seroconversion rate and IMID flare-up or IMID-treatment modality (**Table 2** **/** **Figure 1**). However, subgroup analyses within the group of patient treated with TIMT found a significantly higher odds ratio for N-seroconversion in patients treated with anti-IL12/23 or anti-IL23 (OR 2.64, 95% CI 1.22-5.31, P=0.009). Furthermore, anti-TNF treatment led to significantly lower odds of N-seroconversion compared to patients treated with vedolizumab (OR=0.46, 95%CI 0.22-0.98, P=0.04).

The N-antibody seroconversion rate was significantly higher if the interval between positive PCR and blood withdrawal for serology analysis was shorter than 120 days (RR=4.1, 95%CI 1.64-10.24, P=0.0015).

### 3.3 COVID19 after onset of the vaccination campaign

At the second study sampling timepoint, 1547 of 2065 participants had received complete baseline vaccination (i.e. 2 doses of mRNA-1273, BNT162b2 or ChadOx1 nCoV-19 or 1 dose of JN78436735) and 222 were partially vaccinated. The majority (66.6%) received BNT162b2.

Symptoms suggestive for COVID19 were experienced by 7.6% of patients, positive PCR test was reported by 4.6% and test positivity rate (9.3%) was comparable to before the vaccination campaign. Only seven patients were hospitalized for COVID19 and none required ICU admission. Multiple logistic regression analyses again showed no significant influence of IMID treatment modality on PCR-positivity rate (**Table 2**/**Figure 1**).

An extensive overview of antibody seroconversion rates per vaccine can be found in **Supplementary Table 2**. N-antibody seroconversion was confirmed in 2.6%. IMID flare-up or treatment modality did not significantly influence N-seroconversion rates (**Table 2** **/** **Figure 1**).

**Figure 2:**
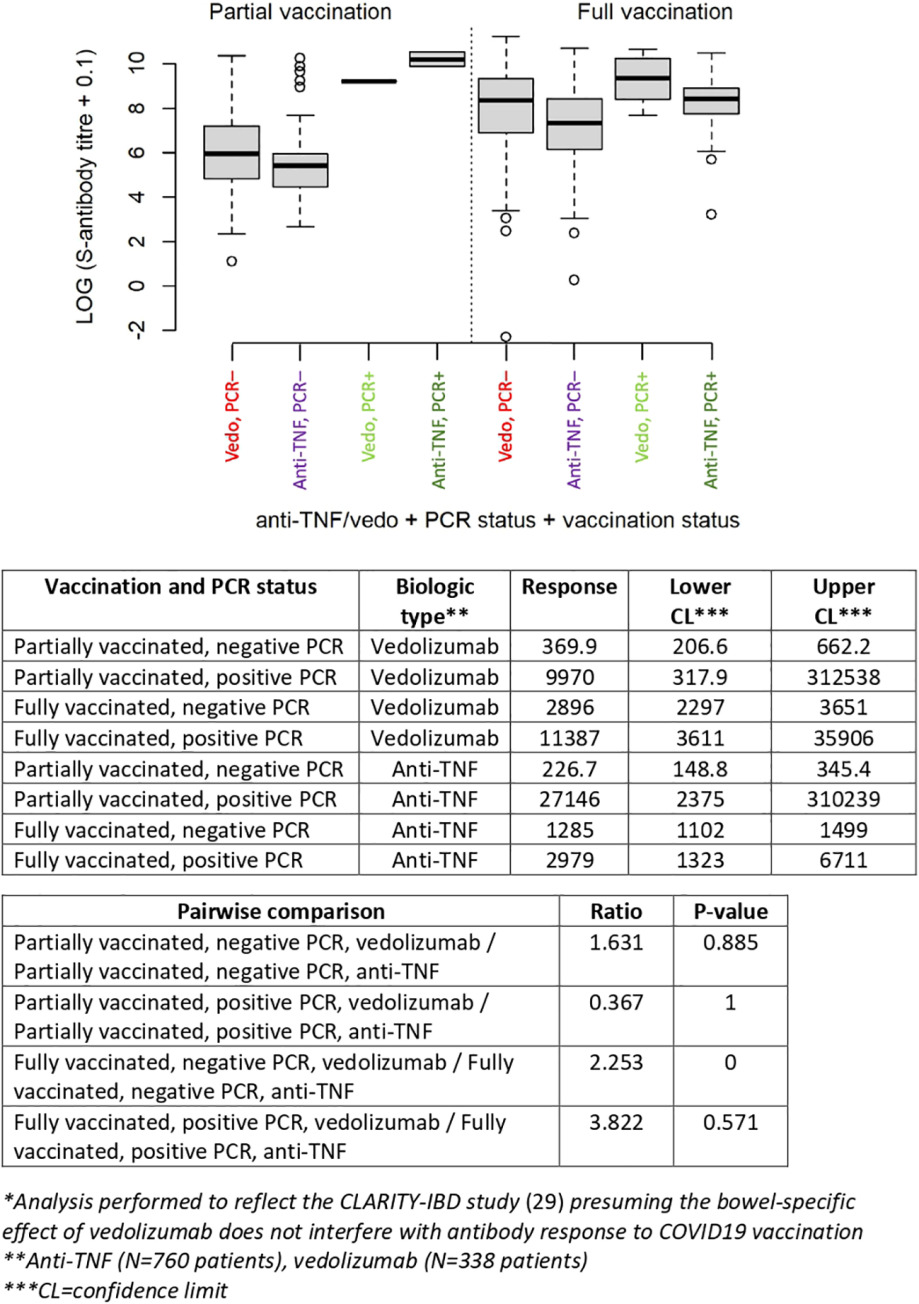
Anti-S-antibody titer (log-transformed) according to vaccination status, PCR status (positive or negative) and treatment modality (anti-TNF or vedolizumab*).

Seroconversion rate for S-antibodies was 91.2%. Active IMID disease significantly decreased the risk of S-seroconversion (RR=0.49, 95%CI 0.36-0.66, P<0.001). In contrast to what was found for N-antibodies, IMID treatment modality interfered with the S-antibody response. The odds for S-seronegativity were significantly higher in patients using TIMT, IMM, combination treatment (IMM+TIMT) and systemic steroids (**Table 2** **/** **Figure 1**). Further subgroup analysis within the patient group treated with TIMT revealed that patients treated with rituximab had a significantly higher odds for S-seronegativity (OR 14.6, 95%CI 4.80-48.2, P<0.001). No significant difference was found when comparing patients treated with anti-TNF to patients on vedolizumab (OR=1.47, 95%CI: 0.84-2.71, P=0.19). However, patients on vedolizumab who received complete baseline vaccination without positive PCR-test, had significantly higher anti-S-antibody titers compared to patients treated with anti-TNF (P<0.001) (**Figure 2**).

In fully vaccinated patients chi square comparisons did not identify significant differences in S-seroconversion rate between different vaccination types (**Supplementary Table 3**).

**Figure 3:**
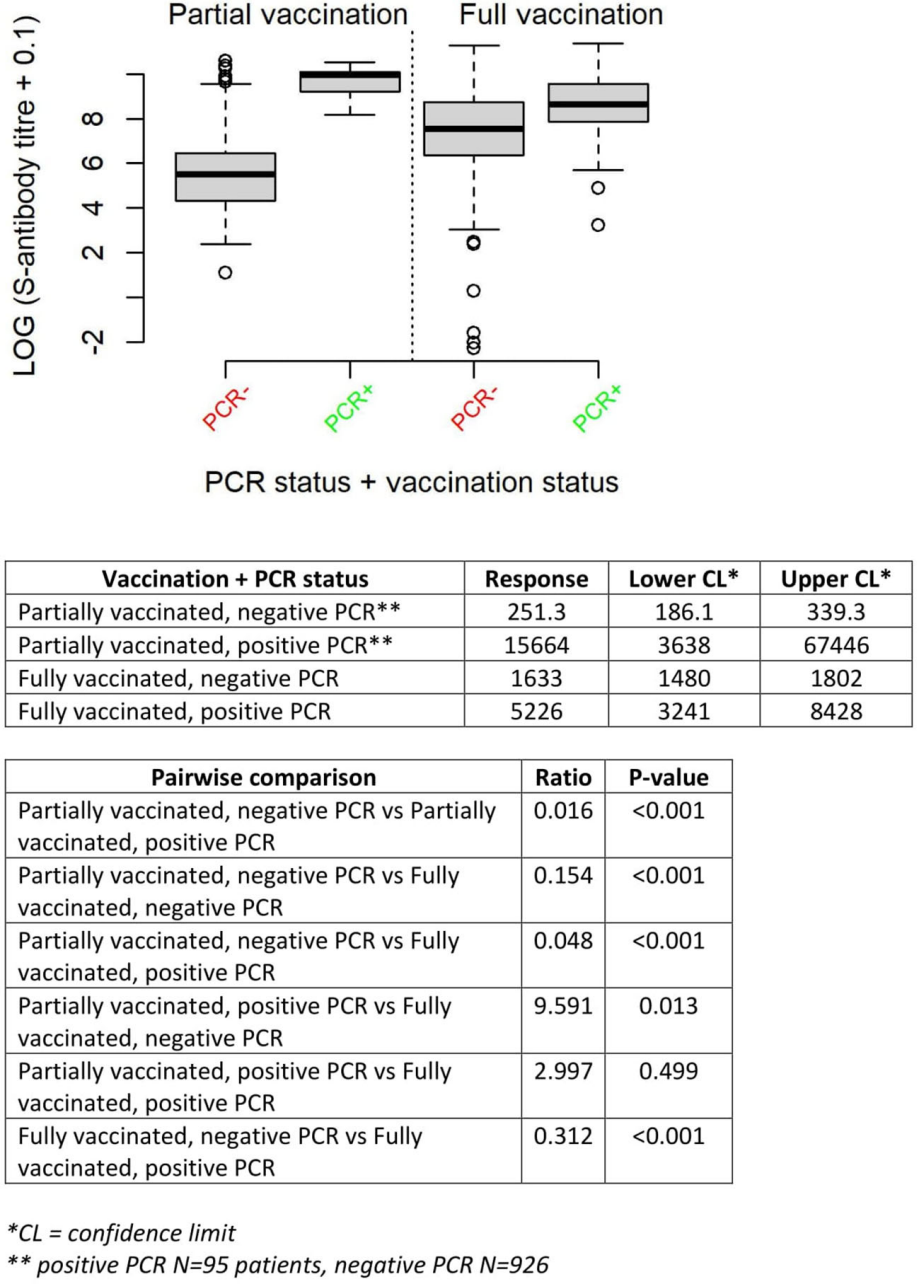
Anti-S-antibody titre (log-transformed) according to vaccination and PCR status.

Pairwise comparisons show that S-antibody titers were higher in patients with previous PCR-positivity compared to patients with equal vaccination status without prior SARS-CoV-2 infection. Furthermore, S-antibody titers were higher in partially vaccinated patients with previous PCR-positivity compared to fully vaccinated patients without positive PCR test (**Figure 3**).

### 3.4 Lower serologic response

A particular subgroup of interest were patients within the lowest quartile of S-antibody titers (see **Supplementary Table 4** for absolute values of S-antibody titers) and those with absence of seroconversion for both S- and N-antibodies following complete baseline vaccination.

Several risk factors for the lowest S-antibody quartile were identified.

Patients vaccinated with ChadOx1 nCoV-19 had higher risk compared to BNT162b2 (RR=2.00, 95%CI 1.70-2.40, P<0.001). Patients with mRNA-1273 vaccination had lower risk compared to BNT162b2 (RR=0.32, 95%CI 0.16-0.67, P=0.001).

Longer interval between vaccination and blood draw led to a significant higher risk of being in the lowest quartile of S-antibody titers (>90 days: RR=1.2, 95%CI 0.95-1.5, P=0.159; >120 days: RR=1.48, 95%CI 1.13-1.95, P=0.01).

Patients with complete baseline vaccination treated with TIMT (OR=2.12, 95%CI 1.46-3.16, P<0.001), combination of TIMT/IMM (OR=2.23, 95%CI 1.57-3.17, P<0.001) or systemic steroids (OR=2.48, 95%CI 1.54-3.97, P<0.001) had significantly higher odds of being in the lowest S-antibody quartile. Among TIMT, treatment with anti-TNF had a higher risk of being in the lowest quartile of S-antibody titer (OR=1.63, 95%CI 1.27-2.08, P<0.001). Rituximab also increased the risk of being in the lowest quartile (OR=6.82, 95%CI 2.31-23.3, P<0.001).

Lastly, patients with active IMID disease (RR=1.30, 95%CI 1.10-1.60, P=0.002) but also active smokers (RR=1.4, 95%CI 1.2-1.7, P=0.002) had a significantly higher risk of being in the lowest quartile of S-antibody titer.

On the other hand, patients with a positive PCR test in the timeframe between first inclusion period and second inclusion had significantly lower risk of ending up in the lowest quartile (RR=0.33, 95%CI 0.15-0.72, P=0.002).

In 66 of 1547 fully vaccinated IMID-patients, no seroconversion for either N- or S-antibodies was found. Following retesting with the highly sensitive and specific LIAISON^®^SARS-CoV-2 TrimericS IgG assay, 3 additional patients with S-seroconversion were picked up, leaving 63 patients (4.07%) with negative serology despite complete vaccination. Interestingly, 90.5% of these combined seronegative patients were treated with a biologic, of which 23 (36.5%) were on anti-TNF and 12 (19%) on rituximab. Furthermore, over half of these patients experienced IMID flare-up since previous inquiry (subgroup demographics in **Supplementary Table 5**).

### 3.5 Impact of SARS-CoV-2 infection and vaccination on IMID disease course

Before vaccination, 39.1% of patients experienced IMID flare-ups 10.7% required systemic steroids. No significant difference in flare-up rate was noted in patients with or without SARS-CoV-2 infection (RR=0.90, 95% CI 0.67-1.20, P=0.553).

At the second BELCOMID inclusion period, after onset of the vaccination campaign, IMID flare-up rate since previous inquiry was 29.1% and 5.7% of patients needed systemic steroids. Patients with positive SARS-CoV-2 PCR or N-antibody seroconversion prior to vaccination, did not experience increased IMID flare-up rates compared to patients without prior COVID19 (RR=1.10, 95%CI 0.72-1.50, P=0.907).

## 4 Discussion

IMID-patients may be more affected by the pandemic compared to healthy peers. Particularly patients taking immunosuppressants expressed increased concern about potential SARS-CoV-2 infection and interactions with their IMID therapy, leading to increased psychological distress and reduced quality of life (17, 18). Previous observational studies showed that IMID-patients have a higher prevalence of COVID19 compared to healthy controls (19, 20). A review of 62 studies including >300.000 patients showed a COVID19 event rate based on PCR of 0.011 and meta-analysis of seven case-controlled studies found that SARS-CoV-2 infection risk in patients with autoimmune diseases including IMIDs was significantly higher compared to controls (OR=2.19; 95%CI 1.05-4.58, P=0.038) (21).

In BELCOMID, SARS-CoV-2 infection prevalence was assessed by combination of reported PCR-positivity and measured N-seroconversion. Prior to the vaccination campaign, prevalence of COVID19 in our patients remained low. Remarkably, N-antibody seroconversion rates were lower than PCR-positivity rates. This observation might have a multifactorial basis. On the one hand it might indicate a potential attenuation of serologic responses to SARS-CoV-2 infection in IMID populations. Indeed, Simon et al. found that IMID-patients receiving cytokine inhibitors, had lower N-seroconversion rates compared to healthy individuals (22). However, in the BELCOMID cohort, multivariate logistic regression showed no clear and consistent impact of TIMT, IMM or their combination on positive PCR rate and N-seroconversion rate over both registration periods. In contrast to what was reported in the review of Akiyama et al. (21), systemic steroids also did not influence N-antibody seroconversion rate.

Another potential explanation for the discrepancy between N-seroconversion and PCR-positivity rate lies in the durability of the humoral response and the relative timing of PCR-testing and blood withdrawal for serologic analysis. Indeed several prospective and observational studies have reported progressive waning of the humoral immune response to SARS-CoV-2 infection and COVID19 vaccination over time (23–29). Following infection, antibody titers have been shown to decline from 8-9 weeks after onset of symptoms with detectable levels up to 12 weeks (30, 31). A large Italian longitudinal prospective study even revealed a 46% decay of S-antibodies in 9 months (32) and the Virus Watch prospective community cohort study in England and Wales showed that N-antibody titers started to decline onwards from 120 days after PCR testing (33). Similarly, in our cohort we demonstrated in non-vaccinated patients that the N-seroconversion rate declined if the interval between positive PCR test and blood withdrawal for N-antibody assessment exceeded 120 days.

Few studies assessed serologic responses to COVID19 vaccination in IMID-patients compared to healthy controls. IMID-patients and particularly patients receiving immunosuppressive treatment were excluded from the registrational COVID19 vaccination trials. Real-world observational data therefore helps to fill this gap in knowledge. A prospective study in patients with acquired or inherited immune disorders showed variable immune responses to vaccination with BNT162b2 (34). A meta-analysis focusing on studies in IMID-patients showed that a significantly smaller proportion of IMID-patients seroconverted after mRNA vaccination compared to healthy controls (OR=0.086; 95%CI 0.04–0.21; P<0.001) (35). In our study the S-antibody seroconversion rate was high (91.2%), but we identified several factors that influenced S-antibody response.

First, we found that TIMT, with or without combined IMM and systemic steroid treatment were associated with significantly reduced S-seroconversion rates and increased odds of being in the lowest S-antibody titer quartile. Subgroup analyses within the TIMT group, revealed anti-TNF and rituximab as risk factors for lower S-titers. This is in line with Garcillàn et al, who found that corticosteroids, B-cell depleting therapies and JAKi substantially affect vaccine immunogenicity (36) and with the VIP-study that revealed lower antibody levels in IBD patients on infliximab with or without thiopurines and tofacitinib (37). Importantly, we confirmed findings from the CLARITY-IBD study, showing that the blunting effect of anti-TNF on S-antibody response is more pronounced than that of the gut-selective vedolizumab (38, 39).

Next, vaccination characteristics seem to impact S-serology. Pairwise comparisons in our broader IMID cohort again confirmed the CLARITY-IBD study findings (39) by demonstrating higher S-antibody titers in partially vaccinated patients with previous PCR-positivity compared to fully vaccinated patients without positive PCR test. Studies comparing efficacy of different vaccination types in IMID-patients are limited. In our cohort, the risk of being in the lowest S-antibody quartile was more pronounced in patients who received ChadOx1 nCoV-19 compared to other vaccine types. This is similar to the findings of a Taiwanese, single-center study reporting higher positive rates of anti-S IgG and higher titers of anti-S IgG after two doses of mRNA-1273 or BNT162b2 compared to ChAdOx1 (40). In a British IBD population, the highest antibody response was also seen in patients receiving BNT162b2 vaccination (41).

Thirdly, just as for the N-antibodies, our results suggest a decline of S-antibody titer over time as an interval of more than 120 days between last vaccine dose and serology analysis led to a significantly increased risk of being in the lowest quartile of S-antibody titers. However, the same time interval was not associated with a significant difference in S-antibody seroconversion rate which might be related to a longer durability of S-antibody response. Indeed other observational and prospective studies have shown a faster and higher rate of decline in N-antibodies compared to S-antibodies (29, 31, 42).

Remarkably, our study identified 63 patients with combined seronegativity after complete vaccination. A potential explanation for this observation could be that this subgroup consist of patients with more refractory IMID disease. This is suggested by the high rate of TIMT and combination of TIMT/IMM use. Furthermore, there is a numerically higher rate of IMID flare-up leading to higher corticosteroid use in this subgroup compared to the total BELCOMID population. Therefore, based on our previously described findings, this refractory subgroup may have been prone to increased attenuation of antibody responses. A third registration timepoint is foreseen within the BELCOMID study protocol. This registration timepoint will enable to assess the interaction between IMIDs and COVID19 after booster vaccination. If absence of seroconversion persists despite booster vaccination, additional analyses including assessment of cellular immunity and genetic predisposition may provide answers to whether these patients actually do mount sufficient protection against SARS-CoV-2.

Another particular finding was the interaction between active smoking and antibody titers. Smoking might facilitate SARS-CoV-2 viral entry through epigenetic mechanisms that alter transcription of several key proteins implicated in the development of COVID19 (43). However, results of observational studies remain controversial. Some studies have shown lower seroconversion rates in active smokers whereas others identified smoking as a potential risk factor for severe COVID19 (44). In our study smokers did have significantly higher risk of being in the lowest quartile of S-antibody titers. This indicates that active smoking negatively impacts humoral response to COVID19 vaccination. The exact mechanism for this phenomenon remains unknown (44).

Our study has some limitations. First, patients were recruited during routine follow-up at their respective treatment facilities. This might have caused recruitment bias by overlooking patients that were, for example, admitted at ICU with severe COVID19 bypassing contact moments with their treating physician. Numbers of patients with severe COVID19 requiring ICU admission in our cohort were however very low. Therefore, we were unable to draw any conclusions towards the association between IMIDs and severe COVID19. Other observational studies however, confirm low rates of severe COVID19 even in unvaccinated IMID cohorts (28, 45). Pooled analysis of 3 large international registries SECURE-IBD, GRA and PsoProtect identified old age, number of comorbidities, use of systemic steroids, thiopurines or combination of anti-TNF with a thiopurine (but not anti-TNF monotherapy), methotrexate, rituximab and JAKi as risk factors for severe COVID19 (36, 46–51). These associations were again found in predominantly unvaccinated populations.

Secondly, evaluation of potential differences in effect on S-antibody response between different IMID types was not performed. This was not within the study goals and not reliably achievable with our statistical models.

Lastly, only humoral response to COVID19 was assessed and we did not report on different strains of SARS-CoV-2. Serological assays may be challenged by different viral variants. However, so far no impairment of the used Abbott^®^ antibody assays targeting the nucleocapsid protein has been reported (52). Furthermore, previous studies have shown good correlation between humoral response and T-cell mediated immunity (53).

Strengths of our study include the multidisciplinary design which may serve as a platform for future multidisciplinary IMID-COVID19 related research initiatives. Furthermore, our study focusses on a spectrum of IMID pathologies taking into account all possible treatment modalities without interference with the routine follow-up and treatment decisions in clinical practice. Both S- and N-antibodies were analyzed and combined seronegativity after vaccination was checked with an additional LIAISON^®^SARS-CoV-2 TrimericS IgG assay. Last but not least, the prospective approach with repeated sampling at different timepoints throughout the several stages of the pandemic enables analysis of intra-patient variations and allows study of the IMID-COVID19 interaction before vaccination, after baseline vaccination and, in the future, after booster vaccination. We therefore believe that our large real-life study cohort can serve as a good representation of IMID-patients in general.

In conclusion, the BELCOMID study prospectively evaluated SARS-CoV-2 infection and vaccination in a real-life cohort of IMID-patients followed at two Belgian tertiary centers, with emphasis on impact of IMID treatment modality and disease course. Our results confirm the benign course of COVID19 in this population and show no significant impact of SARS-CoV-2 infection on IMID disease course.

The blunted antibody response in patients treated with systemic steroids, TIMT and/or IMM and the presumed limited long-term duration of humoral response to both SARS-CoV-2 infection and vaccination stresses the importance of complete and repeated vaccination in IMID-patients. Furthermore, given the lower S-antibody response, this study underlines the importance of smoking cessation in IMID-patients particularly during the pandemic.

## Data availability statement

The raw data supporting the conclusions of this article will be made available by the authors, upon reasonable request.

## Ethics statement

The studies involving human participants were reviewed and approved by the Ethics Committees of University Hospital of Ghent (BC-08030) and University Hospitals of Leuven (S64422). The patients/participants provided their written informed consent to participate in this study.

## Author contributions

Within the BELCOMID study group all members played a substantial role in set-up, design, data collection and data processing. Statistical analyses were done by JG, TL, MT, and SV with support of the Ghent University Biostatistics Unit. Manuscript was drafted by JG and then reviewed and adapted by all members of the BELCOMID study group. All authors contributed to the article and approved the submitted version.
