# Supplementary material for: SARS-CoV-2 infection and COVID19 vaccination across eight immune-mediated inflammatory disorders: A prospective, real-life Belgian cohort study – the BELCOMID study
Source: Front Immunol. 2023 Mar 1;14:1126351. doi: 10.3389/fimmu.2023.1126351 (PMC10014825; doi:10.3389/fimmu.2023.1126351)
Supplement: Supplementary file 1 [file DataSheet_1.pdf]

Extensive description of methodology

Design and goal

During the first peak of the COVID19 pandemic in March-April 2020, a cross-disciplinary consortium was set up between the University Hospitals of Leuven and the Ghent University Hospital, Belgium. Within this consortium a multidisciplinary, prospective, observational cohort study was developed; namely the BELCOMID study. The study was approved by the ethics committee of both University Hospitals (BC-08030/S64422) .

The goal of this study was twofold. The initial aim was to explore the association between COVID19 and IMIDs in a large, real-life population. This includes prospective analysis of exposure to SARS-CoV-2 and COVID19 infection and relating this information to the IMID disease course and type of IMID treatment within the study population. Secondly, as the COVID19 pandemic evolved and national vaccination campaigns were launched, the interaction between COVID19 vaccination and IMIDs was studied in the same real-life patient cohort.

Data collection and sampling

For these purposes, both clinical patient data and blood samples were collected at 3 carefully chosen time periods between December 2020 until February 2022 with a time interval of at least 4 months between sequential sampling (see timeline below):

1) Period 1: prior to the start of the national vaccination campaign (from 17/12/2020 until 28/02/2021)
2) Period 2: prior to the start of the booster vaccination campaign (from 01/07/2021 until 24/09/2021)
3) Period 3: after onset of the booster vaccination campaign (from 03/01/2022 until 15/03/2022)

A pseudonymized electronic case report form (eCRF) was constructed using REDCAP® software. Collected clinical data included patient reported outcomes, clinical information from electronic patient files and SARS-CoV-2 serology results. Patient data comprised information on COVID19 including exposure risk behavior, symptoms and outcomes based on the CORE COVID19 case report form from the International Severe Acute Respiratory and emerging Infection Consortium (ISARIC) (https://isaric.org/research/covid-19-clinical-research-resources/covid-19-crf/), and COVID19 vaccination specifics. Furthermore, IMID-specific symptoms, disease course, treatment and patient reported outcomes (PRO) were registered. For SARS-CoV-2 serologic testing, the Abbott ArchitectTM SARS-CoV-2 immunoglobulin G (IgG) assay[1] on the Architect i2000SR analyser (Abbott, Lake Forest, Illinois, USA) was used to detect anti-nucleocapsid antibodies (N-antibodies) and the Abbott ArchitectTM SARS-CoV-2 IgG II Quant assay[2] on the Architect i2000SR analyser (Abbott, Lake Forest, Illinois, USA)was used to detect anti-spike protein antibodies (S-antibodies) (11)(12). Blood samples of vaccinated patients in whom no seroconversion for both S- and N-antibodies was found,

---

[1] Interpretation of Abbott ArchitectTM SARS-CoV-2 immunoglobulin G (IgG) assay on the Architect i2000SR analyser (Abbott, Lake Forest, Illinois, USA): ≤1.4 Index = negative or intermediate, >1.4 Index = positive

[2] Interpretation of Abbott ArchitectTM SARS-CoV-2 IgG II Quant assay on the Architect i2000SR analyser (Abbott, Lake Forest, Illinois, USA): <50AU/mL = no seroconversion, ≥50AU/mL = seroconversion

were doublechecked with the highly sensitive and specific LIAISON®SARS-CoV-2 TrimericS IgG assay on the Liaison XL (Diasorin S.P.A.,Saluggia, Italy)[3] (13).

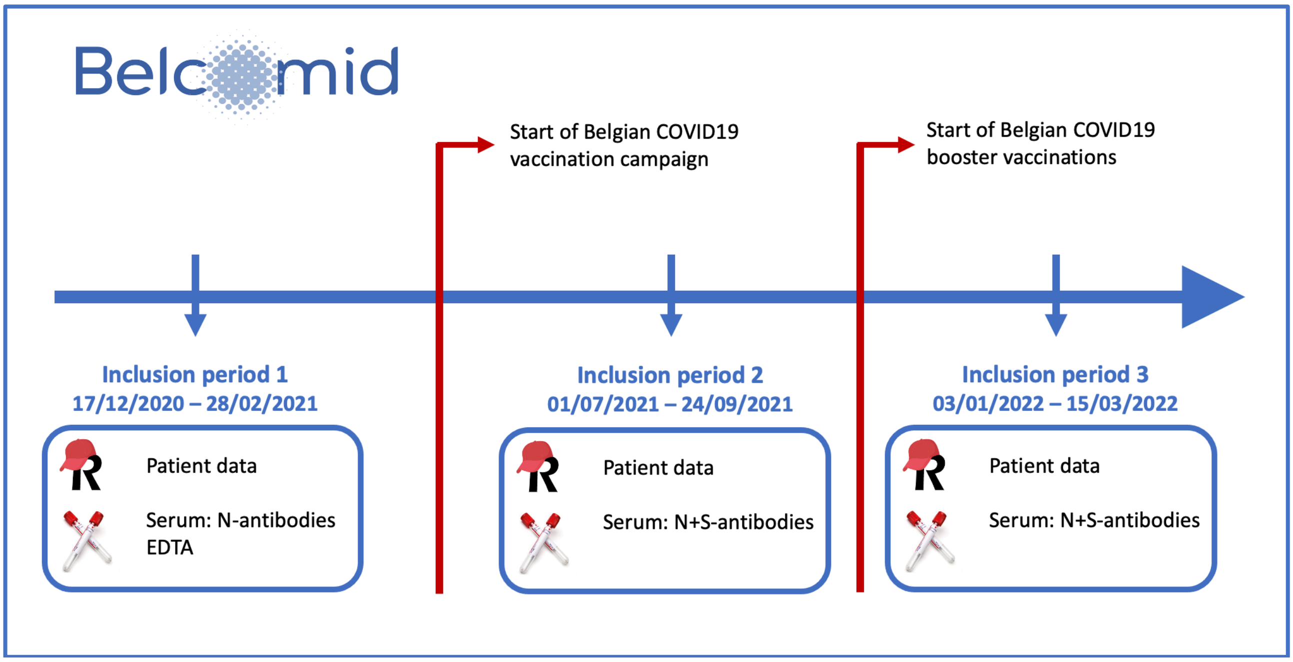

Supplementary Figure 1: BELCOMID sampling timeline

Target population

All patients with IMIDs of the gut (Crohn's disease, ulcerative colitis and indeterminate colitis), joints (rheumatoid arthritis, psoriatic arthritis and spondylarthritis) and skin (psoriasis, hidradenitis suppurativa and atopic dermatitis) seen at the two participating university hospitals were invited to participate at the 3 above described timepoints. Both patients under conventional treatment and/or TIMT were included. Conventional treatment comprised treatment options without immunomodulatory effect (N-IM) and immunomodulating treatment options (IMM). N-IM options included acitretin, metformin, zinc, antibiotics, mesalazine, sulfasalazine, topical creams, topical calcineurin inhibitors and light therapy. IMM included methotrexate, ciclosporin, dimethyl fumarate, mycophenolate mofetil, leflunomide, hydroxychloroquine or thiopurines (azathioprine, 6-mercaptopurin). TIMT options included biologics, JAKi and PDE4 inhibitor apremilast.
No target for sample size was set at the start of the study since the goal was to explore trends in a real-life patient cohort. Therefore, the BELCOMID study was not powered for any of the performed (subgroup) analyses.

Statistical analyses

The two primary endpoints were positive PCR test and SARS-CoV-2 serology reflecting previous SARS-CoV-2 exposure or COVID19 vaccination. Associations of the two endpoints with IMID treatment modality, IMID disease activity and increased SARS-CoV-2 risk were examined. Active disease was a composite variable constructed in de dataset based on

[3] Interpretation of LIAISON®SARS-CoV-2 TrimericS IgG assay on the Liaison XL (Diasorin S.P.A.,Saluggia, Italy): <33.8BAU/mL = no seroconversion, ≥33.8BAU/mL = seroconversion

combination of a multitude of other variables that indicate active disease in the questionnaire such as: need for systemic steroids, hospitalization for flare, change of maintenance medication etc.

Both marginal and conditional associations were examined. The marginal associations were tested using two-sided Pearson's chi-squared tests, not taking into account potential confounders. Conditional effects were tested using adjusted binary logistic regression models and multiple logistic regression models. Due to the limited number of expected events (seroprevalence was estimated at 3.4-6.0%, see above), the number of explanatory variables that could be included in the binary logistic regression models of positive PCR test and high SARS-CoV-2 serology was restricted. Hence, to test the conditional effect of IMID treatment modality in the total population, the binary logistic regression models were adjusted for the propensity score of the respective treatment, increased exposure risk and presence of BMI category. The propensity score was estimated by fitting a logistic regression model where treatment is the response and potential confounders are the predictors.

Potential confounders of the association between treatment and self-reported positive PCR test or SARS-CoV-2 serology status are considered to be: age, gender, smoking status, exposure risk, BMI category, IMID type, comorbidities and vaccination status. For models including infliximab or anti TNF, the propensity score was calculated without taking IMID type into account. For models in subgroups of fully or partially vaccinated patients, the propensity score was calculated without taking vaccination status into account.

For the continuous endpoint S-antibody titer, linear regression analyses were performed.

All hypothesis testing was performed at the 5% significance level. No adjustment for multiple testing was made.

For descriptive statistics SPSS Statistics version 27 was used. Analyses were performed in R version 4.0.2 with support of the Ghent University Biostatistics Unit.

Supplementary Figure 2:

IMID patients experiencing symptoms suggestive of COVID19 per month

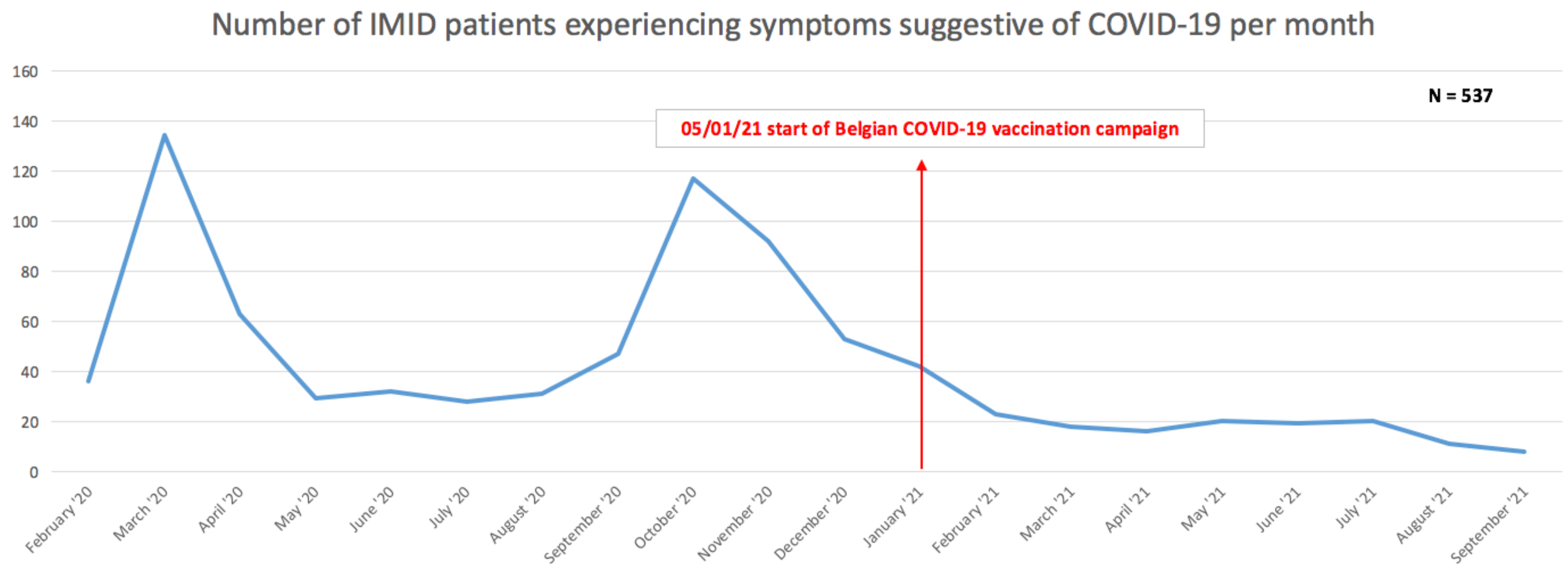

Supplementary Table 1:

Overview of IMID treatment per IMID type and per registration period

	Dermatologic IMID		IBD		Rheumatologic IMID	
	Period 1 (N=316)*	*Period 2* (N=346)*	*Period 1 (N=1344)*	*Period 2 (N=1340)*	*Period 1 (N=505)*	*Period 2 (N=379)*
**TIMT**	206	210	1104	1108	268	234
**IMM**	48	42	143	122	286	231
**N-IM**	50	41	57	38	7	3
**Combination TIMT/IMM**	9	6	111	105	144	137
**Systemic steroids**	20	9	67	34	145	75
**Infliximab**	-	1	471	484	34	33
**Adalimumab**	19	15	132	110	17	16
**Etanercept**	9	8	-	-	94	82
**Certolizumab**	1	1	-	-	12	10
**Vedolizumab**	-	-	329	338	-	-
**Ustekinumab**	15	16	111	110	3	1
**Anti-IL23 (guselkumab, risankizumab, tildrakizumab)**	71	76	-	-	-	-
**Anti-IL17 (ixekizumab, secukinumab, brodalumab)**	64	69	-	-	17	13
**Dupilimumab**	23	18	-	-	-	-
**Tocilizumab**	-	-	-	-	10	11
**Apremilast**	7	8	-	-	10	8
**Tofacitinib**	-	-	17	21	4	4
**Baricitinib**	-	-	-	-	6	6
**Filgotinib**	-	-	-	-	5	5
**Upadacitinib**	-	-	-	-	2	5
**Abatacept**	-	-	-	-	28	22
**Rituximab**	-	-	-	-	38	27
**Antibiotics**	22	13	-	-	-	-
**Acitretin**	24	21	-	-	-	-
**Metformin**	10	10	-	-	-	-
**Zinc**	3	4	-	-	-	-
**Methotrexate**	37	40	47	46	9	27
**Ciclosporin**	22	8	-	-	-	-
**Dimethyl fumarate**	1	1	-	-	-	-
**Mycophenolate Mofetil**	1	-	-	-	-	-
**Thiopurin**	1	-	96	76	1	1
**5-ASA**	-	-	202	181	-	-

**Sulfasalazine**	-	-	21	14	17	11
**Leflunomide**	-	-	-	-	46	33
**Hydroxychloroquin**	-	-	-	-	10	7

* Period 1= before start of national vaccination campaign, Period 2 = before booster vaccination

Supplementary Table 2:

Serology results versus vaccination status/type at 6 months follow-up

		**Serology status**			
		*S+/N+**	*S+/N-*	*S-/N+*	*S-/N-*
**Vaccination status**					
	Fully vaccinated	30 (1.9%)	1274 (80.7%)	-	66 (4.2%)
	Partially vaccinated	4 (0.3%)	117 (7.4%)	-	27 (1.7%)
	Not vaccinated	2 (0.1%)	14 (0.9%)	1 (0.1%)	42 (2.7%)
**Type of vaccine**					
	mRNA-1273 (Moderna)	2 (0.1%)	112 (7.4%)	-	4 (0.3%)
	BNT162b2 (Pfizer-BioNTech)	20 (1.3%)	940 (62.2%)	1 (0.1%)	56 (3.7%)
	ChadOx1 nCoV-19 (Astra Zeneca)	9 (0.6%)	289 (19.1%)	-	25 (1.7%)
	JN78436735 (Johnson & Johnson)	3 (0.2%)	44 (2.9%)	-	6 (0.4%)

* *Abbott ArchitectTM SARS-CoV-2 IgG assay for N-antibodies: ≤1.4 = no seroconversion, >1.4 = seroconversion. Abbott ArchitectTM SARS-CoV-2 IgG II Quant assay for S-antibodies: <50AU/mL = no seroconversion, ≥50AU/mL = seroconversion*

Supplementary Table 3:

Differences in S-seroconversion rate between different vaccination types

**Outcome parameter**	**Compared vaccination types**	**Chi square test**
**No S-seroconversion**	mRNA-1273 vs BNT162b2	RR 0.6, 95% CI 0.22-1.60, P=0.43
	ChadOx1 nCoV-19 vs BNT162b2	RR 1.4, 95% CI 0.88-2.20, P=0.21
	mRNA-1273 vs ChadOx1 nCoV-19	RR 0.4, 95% CI 0.16-1.23, P=0.10

Supplementary Table 4:

S-antibody titres* (absolute values) at inclusion period 2

		Absolute value (AU/mL)
Mean		5322.91
Median		1631.30
Range		88091.70
Minimum		0.00
Maximum		88091.70
Quartiles	**25%**	385.60
	**50%**	1631.30
	**75%**	5952.40

* Abbott Architect™ SARS-CoV-2 IgGII Quant assay (≥50AU/mL=seroconversion)

Supplementary Table 5:

Patients with negative S- and N-serology after complete baseline vaccination

**Demographic data**		**N=63**
Age	<60 years old	47.6%
	>/=60 years old	47.6%
Gender	Male	50.8%
	Female	49.2%
IMID type	Dermatologic IMID<br>Hidradenitis suppurativa<br>Psoriasis<br>Atopic dermatitis	11.1%<br>1 patient<br>5 patients<br>1 patient
	IBD<br>Crohn's disease<br>Ulcerative colitis<br>IBD type unclassified	46.0%<br>19 patients<br>8 patients<br>1 patient
	Rheumatologic IMID<br>Rheumatoid artritis<br>Spondyloarthritis	42.9%<br>26 patients<br>1 patient
Smoking status	Active smoker	19.0%
	Non-smoker	73.0%
BMI	Obese (≥30kg/m^2)	29.1%
Comorbidities	Diabetes	11.1%
	None	20.6%
Active IMID disease	Yes	58.7%
Increased exposure risk	Yes	41.3%
PCR test	Yes, positive	1.6% (=1 patient)
	Yes, negative	42.9%
Symptoms suggestive of COVID19	Yes	3.2%
Hospitalisation for COVID19	Yes	1.6%
IMID Treatment	TIMT<br>Anti-TNF<br>Vedolizumab<br>Guselkumab<br>Dupilimab<br>Tocilizumab<br>Tofacitinib<br>Upadacitinib<br>Rituximab	90.5%<br>23 patients<br>8 patients (IBD)<br>3 patients (dermato)<br>1 patient (dermato)<br>1 patient (rheumato)<br>1 patient (IBD)<br>1 patient (rheumato)<br>12 patients (rheumato)
	IMM	50.8%
	Combination TIMT/IMM	47.6%
	N-IM	14.3%
	Systemic steroids	27.0%
Vaccine type	*mRNA-1273 (Moderna)*	4.8%

	BNT162b2<br>*(Pfizer-BioNTech)*	59.7%
	ChadOx1 nCoV-19<br>*(Astra Zeneca)*	25.8%
	JN78436735<br>*(Johnson & Johnson)*	9.7%
